# Amniotic fluid stem cells alleviate neuronal injury-related changes in spina bifida by inducing autophagy via regulating the FOXO1/STAT3 axis

**DOI:** 10.1080/15476278.2026.2728258

**Published:** 2026-09-11

**Authors:** Cuicui Li, Hepu Lin, Hongwei Gao, Ning Liu, Yiwu Dai, Yongchun Luo

**Affiliations:** a Department of Neurosurgery, The First Medical Center of the PLA General Hospital, Beijing, China; b Department of Neurosurgery, The Seven Medical Center of the PLA General Hospital, Beijing, China

**Keywords:** Amniotic fluid stem cells, autophagy, FOXO1, neural stem cells, spina bifida

## Abstract

Spina bifida (SB) is associated with progressive spinal cord injury and limited therapeutic options for neural protection. Amniotic fluid stem cells (AFSCs) have shown regenerative potential, but their effects on SB-related neuronal injury and the underlying mechanisms remain unclear. In this study, we investigated whether AFSCs alleviate neuronal injury-related changes in SB rats through the modulation of autophagy via the FOXO1/STAT3 pathway. SB-like lesions were induced in neonatal rats, followed by the intramedullary injection of AFSCs or vehicle on postnatal day 1. Behavioral assessment was performed using Basso, Beattie, and Bresnahan locomotor scoring on days 2, 7, and 14 after injection, and lesion-centered lumbosacral spinal cord tissues were collected on day 15. AFSC treatment partially improved hindlimb locomotor scores and reduced neuronal injury-related alterations in SB rats. AFSCs also increased autophagy-associated markers, including LC3II/I and Beclin1, and reduced p62 mRNA expression in vivo and in neural stem cells isolated from SB rats. Mechanistically, AFSC treatment was associated with reduced FOXO1 and enhanced STAT3 signaling. FOXO1 overexpression or STAT3 inhibition attenuated AFSC-mediated effects, whereas siRNA-mediated FOXO1 knockdown enhanced these effects; combined STAT3 knockdown partially reversed the effects of FOXO1 silencing. These findings suggest that AFSCs alleviate SB-related neuronal injury-associated changes by regulating autophagy through the FOXO1/STAT3 axis. This study provides preclinical evidence for an experimental neuroprotective mechanism of AFSCs, although further validation is required before translational applicability can be considered.

## Introduction

Spina bifida (SB) is a common and severe congenital malformation, caused by incomplete closure of the neural tube during neuroembryonic development. The incidence rate in the population is between 1‰ and 2‰, varying among different populations, with China being a high-incidence area.^1^ SB is a polygenic hereditary disease resulting from the combined effects of genetic and environmental factors.^2^ It is typically characterized by the protrusion of the spinal cord outside the spine, forming a meningocele. Patients often accompany motor and sensory neurological dysfunctions, which may lead to lower limb weakness or paralysis, as well as an increased risk of pressure sores due to sensory loss.^3^ Currently, no single medical or surgical treatment can fully improve or restore the neurological dysfunctions associated with SB and meningocele.^4^ Prenatal surgical repair has been shown to improve selected outcomes compared with postnatal repair, including reduction of hindbrain herniation and a decreased need for cerebrospinal fluid shunting in appropriately selected patients; however, neurological deficits may persist, and the procedure is associated with maternal–fetal risks. Therefore, studying the pathogenesis of SB and developing corresponding therapeutic strategies is of great significance for reducing birth defects and improving the quality of the population.

Neural stem cells (NSCs) are a class of cells with self-renewal and differentiation capabilities, playing an important role in the repair of the nervous system.^5^ Autophagy is an important intracellular quality control and repair mechanism that maintains the homeostasis of stem cells by clearing dysfunctional organelles and protein aggregates, and participates in self-renewal, differentiation, and reprogramming of cells during development.^6^ Autophagy plays a role in the generation and development of NSCs, and even in cell fate determination.^7^ In the context of neurological dysfunction caused by spinal cord injury, the activation of autophagy can enhance the efficacy of NSCs derived from induced pluripotent stem cells (iPSCs).^8^ Additionally, exosomes derived from NSCs contribute to the recovery of spinal cord function after spinal cord injury by promoting angiogenesis.^5^ Therefore, the role of autophagy in NSCs in neurological dysfunction caused by SB may be manifested by promoting the proliferation and differentiation of NSCs, as well as reducing neuroinflammation and apoptosis, thereby aiding in the recovery of neurological function.^9^ These findings provide new strategies and potential therapeutic targets for alleviating SB-related neural injury.

Amniotic fluid stem cells (AFSCs) are a class of multipotent stem cells present in amniotic fluid.^10^ They are capable of differentiating into cell types from all three germ layers, including adipocytes, osteoblasts, muscle cells, endothelial cells, neural cells, and hepatocytes.^11^ AFSCs possess low immunogenicity, are easy to culture, have strong proliferation capabilities, stable genomes, non-tumorigenicity, are suitable for genetic modification, and raise no ethical issues.^12^ In the recovery of neurological function after spinal cord injury, hAESCs alleviate spinal cord tissue damage, significantly increase the number of neurons, neurofilaments, and myelin sheaths, and significantly decrease spinal cord cavities and glial scars.^13^ Moreover, AFSCs can promote neurogenesis by secreting various growth factors (GFs), such as brain-derived neurotrophic factor (BDNF), which increases cell proliferation, neurogenesis, cell survival, and myelin synthesis while reducing apoptosis, glial scar formation, and demyelination in spinal cord injury models.^14^ Wei et al. research indicates that the transplantation of intra-amniotic mesenchymal stem cells can improve the amniotic fluid environment in rats with open SB aperta, which is beneficial for the repair of SB.^15^ A systematic review and meta-analysis of preclinical fetal myelomeningocele models further summarized that most stem cell-based prenatal studies used mesenchymal stem cells, mainly in rodent and ovine models, and that intra-amniotic injection of allogeneic amniotic fluid-derived mesenchymal stem cells in rat myelomeningocele models did not compromise fetal survival and was associated with a higher rate of complete defect coverage compared with saline injection.^16^ In a retinoic acid-induced fetal rat myelomeningocele model, intra-amniotic administration of human AFSCs significantly reduced the exposed spinal cord area, decreased neurodegeneration and astrogliosis, and promoted neural protection partly through hepatocyte growth factor secretion by engrafted or amniotic fluid-resident hAFSCs.^17^ These findings indicate that AFSC-based fetal therapy may protect exposed spinal tissue through both physical lesion coverage and paracrine neurotrophic mechanisms. In addition to intra-amniotic approaches, local spinal cord delivery of amniotic-derived stem cells or their conditioned medium has also been investigated in basic spinal cord injury models. Human amniotic fluid-derived mesenchymal stem cells and their conditioned medium improved locomotor recovery, increased endogenous DCX-positive neuroblast generation, and reduced GFAP-positive astrocytic responses after spinal cord injury, supporting the contribution of juxtacrine and paracrine mechanisms to neural repair.^18^ However, safety considerations remain important, as intraparenchymal transplantation of clonally expanded rat amnion mesenchymal stem cells into contused spinal cords failed to improve behavioral recovery and induced aberrant chondrocytic differentiation with excessive extracellular matrix deposition.^19^ Together, these studies provide an important context for evaluating the therapeutic potential and mechanistic basis of AFSC administration in spinal neural defects. Although intra-amniotic injection of AFSCs has shown the ability to promote skin coverage and neural protection in fetal rodent SB/MMC models,^20^ this delivery route may have limited reparative efficiency for deeper spinal cord tissues because transplanted cells and secreted factors are distributed within the amniotic cavity rather than being concentrated within the injured neural parenchyma. Neurological deterioration in SB is considered to arise from direct exposure of the spinal cord to amniotic fluid and subsequent progressive degeneration, which provides a pathological basis for targeting the exposed and damaged spinal cord itself.^21^ Therefore, direct intraspinal injection was selected in the present study to deliver AFSCs precisely to the lesion region, increase local cell retention, and maximize the paracrine and cell-mediated neuroprotective effects within the affected spinal cord tissue. This local delivery strategy is not intended to replace established prenatal surgical closure; instead, it may serve as an experimental adjunctive concept during or in combination with surgical repair, providing neuroprotection and regenerative support at the cellular level while anatomical closure prevents further mechanical and chemical injury.^22^ Nevertheless, the clinical applicability of direct spinal cord delivery of AFSCs remains uncertain and requires rigorous evaluation in clinically relevant fetal repair models before translational feasibility can be considered. However, it is currently unclear whether AFSCs are beneficial for alleviating SB-related neuronal injury and modulating repair-associated molecular pathways. This study assessed the impacts of AFSCs on NSCs autophagy to evaluate their role in alleviating neuronal injury-related molecular and cellular alterations following SB.

## Materials and methods

### The isolation of rat AFSCs

Male and female SD rats (7–9 weeks) were achieved from Vital River (Beijing, China). After mating, pregnant female rats were collected, and on the 14th day of gestation, pregnant female rats were overdosed with anesthesia and then euthanized. The uteri were removed, and each pregnant sac was opened from the anti-mesometrial side to avoid bleeding in the placental area. Amniotic fluid was extracted from each sac using a 30-gauge calibrated needle. The amniotic fluid was then centrifuged at 1400 × *g* for 5 min to pelletize cells. The cell pellets were cultured in 60 mm culture dishes containing 5 ml of low-glucose DMEM supplemented with 10% fetal bovine serum (FBS) and 1% penicillin/streptomycin. The cells were incubated in a humidified incubator at 37 °C with 5% CO_2_ and observed daily under a phase-contrast inverted microscope. The culture medium was changed every 2 to 3 d. The cells were passaged after they reached 80% confluence using 0.25% (w/v) trypsin/ethylenediaminetetraacetic acid (EDTA) and were then cultured in T75 cm^2^ flasks, which were isolated AFSCs.

### The identification of isolated AFSCs

The isolated cells were resuspended in PBS buffer containing antibodies against CD45 (ab27287), CD90 (ab255), and OCT4 (ab181557, Abcam, USA), with a dilution ratio of 1:200, and incubated at room temperature for 30 min to label the cell surface antigens. After incubation, the cells were washed with PBS and then resuspended in 1 mL of PBS buffer, followed by analysis using a flow cytometer (NovoCyte, Agilent, USA) with a laser excitation wavelength set at 488 nm to collect the fluorescence signals of CD45, CD90, and OCT4, respectively.

### The establishment of SB rats

Female rats were mated overnight with male rats. The presence of a vaginal plug the following morning after mating was considered as embryonic day 0 (E0). At E10, spina bifida was induced by a single oral administration of ATRA (40 mg/mL in olive oil; 150 mg per kilogram of body weight). The control rats were injected with olive oil without ATRA at a volume of 4 mL/kg body weight on the same day. After spontaneous delivery, neonatal rats were examined for the presence of an open lumbosacral spinal defect. Newborn rats with visible SB/MMC-like lesions were included in the SB model groups, whereas littermates without malformations from control dams were used as controls.

### Grouping of animal studies

Animals were divided into three groups (*n* = 6/group): Control, Model, and Model+AFSCs. Neonatal SB rats with visible SB/MMC-like lesions were randomly assigned to the Model or Model+AFSCs group using a random-number method, while control animals were randomly selected from litters of dams receiving olive oil alone. Investigators responsible for behavioral scoring and subsequent quantitative analyses were blinded to group allocation. All interventions were performed postnatally on postnatal day 1 (P1), within 24 h after birth. In the Model+AFSCs group, SB rats were intraspinally injected with 1 × 10^7^ AFSCs suspended in 50 μL DMEM at the center of the exposed lumbosacral spinal lesion using a microsyringe under sterile conditions. In the Model group, SB rats were intraspinally injected with 50 μL blank DMEM at the same anatomical site and at the same postnatal time point. No treatments were performed on the animals in the control group. The animals were sacrificed 14 d after injection, on postnatal day 15 (P15), for spinal cord tissue collection. For each SB rat, only the lesion-centered spinal cord segment containing the SB/MMC defect and approximately 2 mm of adjacent rostral and caudal spinal cord tissue was dissected and processed for subsequent assays. The entire spinal cord was not used. For control rats, anatomically matched lumbosacral spinal cord segments were collected to ensure comparable tissue sampling among the groups. Technical success of the injection was defined as completion of the planned intraspinal administration with delivery of the full 50 μL AFSC suspension or vehicle into the lesion-centered lumbosacral spinal cord region, without obvious fluid leakage, severe bleeding, or immediate procedure-related death. After injection, the animals were monitored daily until tissue collection for survival, local bleeding, wound infection, abnormal swelling, and general condition.

### Behavioral assessment

Locomotor performance was evaluated using the Basso–Beattie–Bresnahan (BBB) locomotor rating scale. BBB scoring was performed on days 2, 7, and 14 after AFSC or vehicle injection. The score ranges from 0 to 21, with higher scores indicating better hindlimb locomotor function. The animals were placed individually in an open-field arena and allowed to move freely during the observation period. Each animal was observed for 4 min by two blinded investigators, and the mean score was used for analysis. Scoring was performed by investigators blinded to group allocation.

### RT-PCR assay

RNA was extracted from cells or spinal cord tissues harvested from the lesion-centered lumbosacral segments described above using the TRIzol method, and cDNA was synthesized by reverse transcription with the HiFiScript cDNA Synthesis Kit (CW2569M, CWBIO, China). qRT-PCR amplification was performed using the Hieff® qPCR SYBR Green Master Mix (11201ES03, YEASEN, China) to analyze the levels of relevant genes. The results were analyzed using the 2^−^^ΔΔCT^ method for data analysis, and the sequences of the primers were shown in Table 1.

**Table 1.** Sequences of primers applied in RT-PCR assay.

Gene	Forward primer	Reverse primer
Bax	AGGATGGCTGGGGAGACACCTG	CATGGGCTGGACACTGGACTTC
Bcl-2	CGGGAGATCGTGATGAAGTACATC	ACAACATCGCTCTGTGGATGACTG
p62	GCACTGGCTTTAGGTGTCATA	TTTGTCTCTGCGGGGTTAAGAG
FOXO1	GGCTAACCCGTAGCAAGTGT	CTCAGGCAAGCCCTAGGAAG
STAT3	TGTCCAATGCTGCTGATCGT	TCTCTTTCAAGCTCCGGCTG
β III Tubulin	CTCAGGGGCCTTTGGACATC	CAGGCAGTCGCAGTTTTCAC
MAP2	AGCTAGAGAGCCAGAGAGCC	CCCAATCAATGCTTCCTCGG
SYNAPSIN1	CCAATCTGCCGAATGGGTACA	GCGTTAGACAGCGACGAGAA
Ngf	GGGCCCATTAACGGCTTTTC	CATTGCTCTCTGTGTGGGGT
Egf	GAAGCATTGGACAAGTATGCAT	CAGCTTCTGAGTCCTGTAGTAG
Igf-1	GTGGACCGAGGGGCTTTTACTTC	TTTGCAGCTTCGTTTTCTTGTTTG
Fgf2	ATGGCTCCCTTAGCCGAAGT	AGGAAATGCGAACCCACCTG
Fgf10	TTTGGTGTCTTCGTTCCCTGT	TAGCTCCGCACATGCCTTC
Bdnf	CAACGAAGAAAACCATAA	GTGACCCACTCGCTAATA
Tgf-beta	AACATGATCGTGCGCTCTGCAAGTGCAGC	AGGACGGACAGACGTGATAAGGAA
GAPDH	CTTGTGACAAAGTGGACATT	GCGGAAGGGGCGGAGATGAT

### Western blot analysis

Proteins were obtained from cells or spinal cord tissues harvested from the lesion-centered lumbosacral segments described above using the lysis buffer. The BCA protein assay kit (pc0020, Beyotime, China) was used to detect protein concentration. A 10% SDS-PAGE was prepared for protein electrophoresis. After transferring to a PVDF membrane, the PVDF membrane was blocked with 5% milk for 2 h, then incubated with primary antibodies against Bax (ab32503, 1:800, Abcam, USA), Bcl-2 (ab182858, 1:1000, Abcam, USA), β III Tubulin (ab18207, 1:800, Abcam, USA), MAP2 (ab5392, 1:1000, Abcam, USA), SYNAPSIN1 (ab254349, 1:1000, Abcam, USA), LC3II/I (ab51520, 1:800, Abcam, USA), Beclin1 (ab207612, 1:1000, Abcam, USA), FOXO1 (2880, 1:800, CST, USA), p-STAT3 (9145, 1:1000, CST, USA), STAT3 (30835, 1: 1000, CST, USA), and GAPDH (ab8245, 1:2000, Abcam, USA) at 4 °C overnight. After washing the membrane, the secondary antibody (ab288151, 1: 4000, Abcam, USA) was incubated for 2 h. The LAS 500 imaging system was used for exposure and imaging, and the grayscale values were analyzed using Image J.

### The isolation of rat NSCs from spinal cord tissues

The healthy and SB immature rats were weighed and injected with sodium phenobarbital for anesthesia. NSC isolation was performed using rats sacrificed on P15, corresponding to 14 d after AFSC or vehicle injection in the SB groups. Following this, the spinal cord was dissected and digested into a single-cell suspension using 0.125% trypsin. For SB rats, the dissected tissue consisted of the lesion-centered lumbosacral spinal cord segment containing the SB/MMC defect and approximately 2 mm of adjacent rostral and caudal tissue. For healthy control rats, anatomically matched lumbosacral spinal cord segment was collected. The entire spinal cord was not used for NSC isolation. Then, NSCs were cultured in a complete NSC medium (DMEM/F12) supplemented with 2% B27, 10 ng/ml EGF, 10 ng/ml bFGF, and 100 U of penicillin/streptomycin. The culture was maintained under conditions of 5% CO_2_ and at 37 °C. After five days of culture, the neurospheres were dissociated and seeded onto 96-well plates. Subsequently, the sub-cloned neurospheres were passaged three times to obtain neurospheres derived from single primary cells for subsequent experiments. NSCs isolated from healthy immature rats were named as NC-NSCs, while NSCs isolated from SB immature rats were named as M-NSCs.

### Identification of isolated NSCs

The extracted NC-NSCs and M-NSCs were identified for being Nestin-positive cells using immunofluorescence, with PC12 cells serving as a negative control (LHY3027, ATCC, USA). The extracted cells were seeded into culture dishes that had been pre-placed with treated cover slips, and the cover slips were removed once the cells were close to forming a monolayer. Subsequently, the cells were covered with a 4% neutral formaldehyde fixative and fixed for 15 min. After fixation, they were blocked with 5% goat serum and incubated in a 37 °C humidified incubator for 30 min. The Nestin antibody (1:500, ab105389, Abcam, USA) was diluted in an antibody diluent containing 1× TBS, 5% goat serum, and 0.3% Triton™ X-100, and incubated overnight at 4 °C. The following day, the samples were allowed to warm to room temperature for 15 min and then incubated with the secondary antibody (1:500, ab150077, Abcam, USA) in the dark at room temperature for 1 h. The nuclei were stained with a 1 mg/mL DAPI working solution and incubated in the dark at room temperature for 10 min, after which an anti-fluorescence quenching mounting medium was added. The cells were observed and imaged under a fluorescence microscope (Ts2-FC, Nikon, Japan).

### The co-culture system of AFSCs and NSCs in Transwell

To evaluate influences of AFSCs on NSCs, a co-culture system was established. On the upper chamber of the Transwell, AFSCs were loaded, while in the lower chamber of the Transwell, NSCs were loaded.

### Flow cytometry for the apoptosis analysis

Cells were seeded into a 6-well plate, and after 24 h, once the cells had fully adhered to the plate, they were digested and neutralized with complete culture medium. The cells were then collected by centrifugation at 1000 rpm to form a cell pellet. The cell pellet was washed three times with ice-cold PBS for three minutes each time, and then resuspended in 500 μL of binding buffer. Subsequently, 5 μL of PI dye was added, and the cells were incubated in the dark at room temperature for 15 min. The number of PI-positive cells was detected using a flow cytometer (NovoCyte, Agilent, USA).

### Transfection

The overexpression plasmid for FOXO1 (FOXO1 OE) was constructed, with the empty vector (EV) serving as a negative control. M-NSCs were transfected with the adenovirus containing FOXO1 OE for 48 h. For gene-silencing experiments, M-NSCs were transfected with siRNA targeting FOXO1 (si-FOXO1), siRNA targeting STAT3 (si-STAT3), or a non-targeting negative control siRNA (si-NC). The siRNAs were synthesized by GenePharma (Shanghai, China), and transfection was performed using Lipofectamine 3000 reagent (Invitrogen, Thermo Fisher Scientific, USA) according to the manufacturer's protocol. In selected experiments, si-FOXO1 and si-STAT3 were co-transfected to evaluate whether STAT3 knockdown modified the effects associated with FOXO1 silencing. After 48 h of transfection, the cells were used for co-culture experiments and subsequent RT-PCR analysis. The efficiency of FOXO1 overexpression and the knockdown efficiencies of FOXO1 and STAT3 were verified by RT-PCR analysis.

### Statistical analysis

Data were statistically analyzed using GraphPad 8.0 software. The quantitative data were represented as x ± s. Comparisons between two groups were conducted using Student's *t*-test. Comparisons among multiple groups were performed using one-way ANOVA, and further pairwise comparisons were made using the LSD-t test. BBB locomotor scores measured at multiple time points were analyzed using two-way repeated-measures ANOVA followed by post hoc multiple comparisons. A *p*-value of less than 0.05 was considered to indicate a statistically significant difference.

## Results

Technical success and procedural safety of AFSC administration: All animals assigned to the Model and Model+AFSCs groups successfully received the planned intraspinal injection on postnatal day 1. The technical success rate was 100% for both vehicle injection and AFSC administration. No immediate injection-related death occurred, and all the animals survived until the scheduled tissue collection on postnatal day 15. During the post-injection observation period, no severe procedure-related complications, including persistent bleeding, wound infection, marked local swelling, or deterioration requiring early euthanasia, were observed.

### Influences of AFSCs on locomotor performance, apoptosis, neuronal injury-related markers, and nerve growth factor production in SB rats

**Figure 1.**
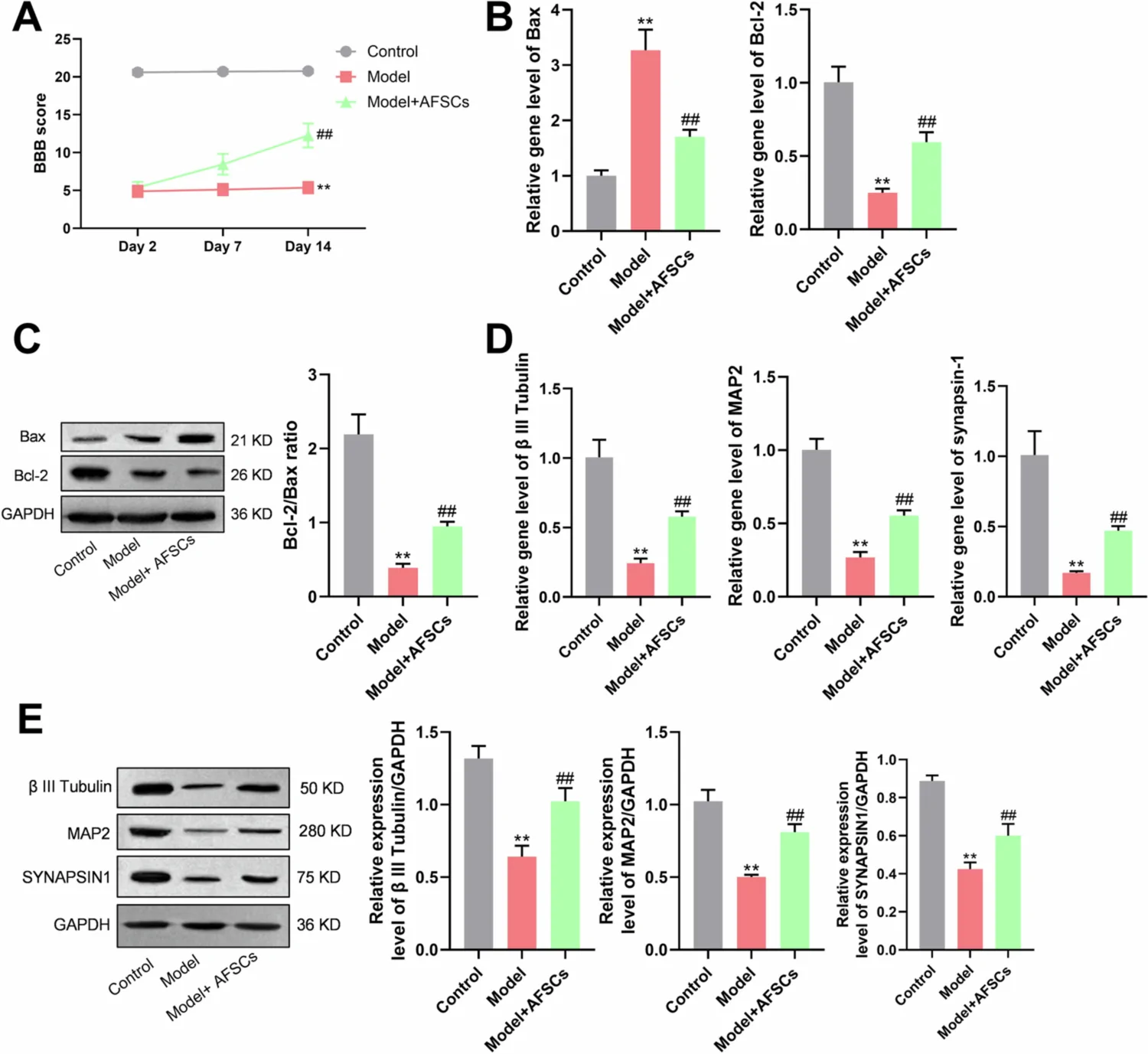
Influences of AFSCs on locomotor performance, apoptosis and neuronal injury-related markers in SB rats. Animals were divided into three groups: Control, Model, and Model+AFSCs. (A) BBB locomotor scores were assessed on days 2, 7, and 14 after AFSC or vehicle injection. (B) Gene levels of Bax and Bcl-2 in spinal cord tissues were detected by RT-PCR. (C) Protein levels of Bax and Bcl-2 in spinal cord tissues were determined by Western blot analysis. (D) Gene levels of β III Tubulin, MAP2, and SYNAPSIN1 in spinal cord tissues were detected by RT-PCR. (E) Protein levels of β III Tubulin, MAP2, and SYNAPSIN1 in spinal cord tissues were determined by Western blot analysis (**p < 0.01 vs. Control, ^##^ *p* < 0.01 vs. Model).

Firstly, isolated AFSCs were identified by flow cytometry, with relatively low expressions of CD45 and high expressions of CD90 and OCT4 (Figure S1). In animal studies, three groups were divided: Control, Model, and Model+AFSCs. BBB locomotor scores were markedly decreased in SB model rats compared with control rats. AFSC-treated SB rats showed a gradual increase in BBB scores, with a significant improvement observed on day 14 compared with untreated SB model rats (Figure 1A). In spinal cord tissues, Bax levels were markedly elevated, while Bcl-2 levels were notably reduced in the Model group, which were remarkably reversed by AFSCs (Figure 1B and C). Furthermore, expressions of the neuronal function related proteins, β III Tubulin, MAP2, and SYNAPSIN1, in spinal cord tissues were sharply diminished in SB rats, which were strikingly enhanced by AFSCs (Figure 1D and E). In addition, the gene levels of nerve growth factors, including Ngf, Egf, Igf, Fgf2, Fgf10, Bdnf, and Tgf-β, in spinal cord tissues were remarkably reduced in the Model group, which were sharply elevated by AFSCs (Figure S2).

### AFSCs enhanced autophagy in spinal cord tissues of SB rats

**Figure 2.**
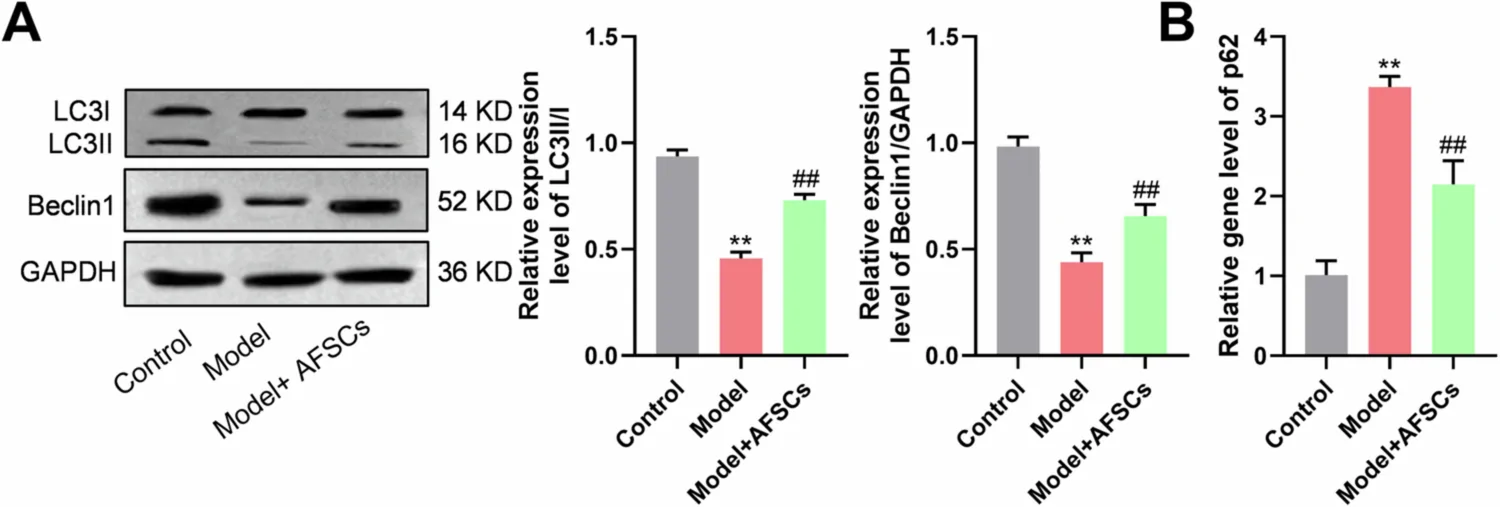
AFSCs enhanced autophagy in spinal cord tissues of SB rats. Animals were divided into three groups: Control, Model, and Model+AFSCs. (A) Western blot analysis was utilized to determine levels of LC3II/I and Beclin1 in spinal cord tissues. (B) Gene level of p62 in spinal cord tissues was detected by RT-PCR (**p < 0.01 vs. Control, ^##^ *p* < 0.01 vs. Model).

In the spinal cord tissues of SB rats, LC3II/I and Beclin1 were strikingly downregulated, expressions of which were notably elevated by the administration of AFSCs (Figure 2A). In parallel, p62 expression was markedly increased in SB spinal cord tissues and was reduced after AFSC administration (Figure 2B). These coordinated changes in LC3II/I, Beclin1, and p62 further supported that AFSCs modulated autophagy-associated molecular alterations in SB spinal cord tissues, implying that the protective effects of AFSCs on neuronal injury-related changes in SB rats might be associated with facilitated autophagy.

### AFSCs regulated the FOXO1/STAT3 signaling in spinal cord tissues of SB rats

**Figure 3.**
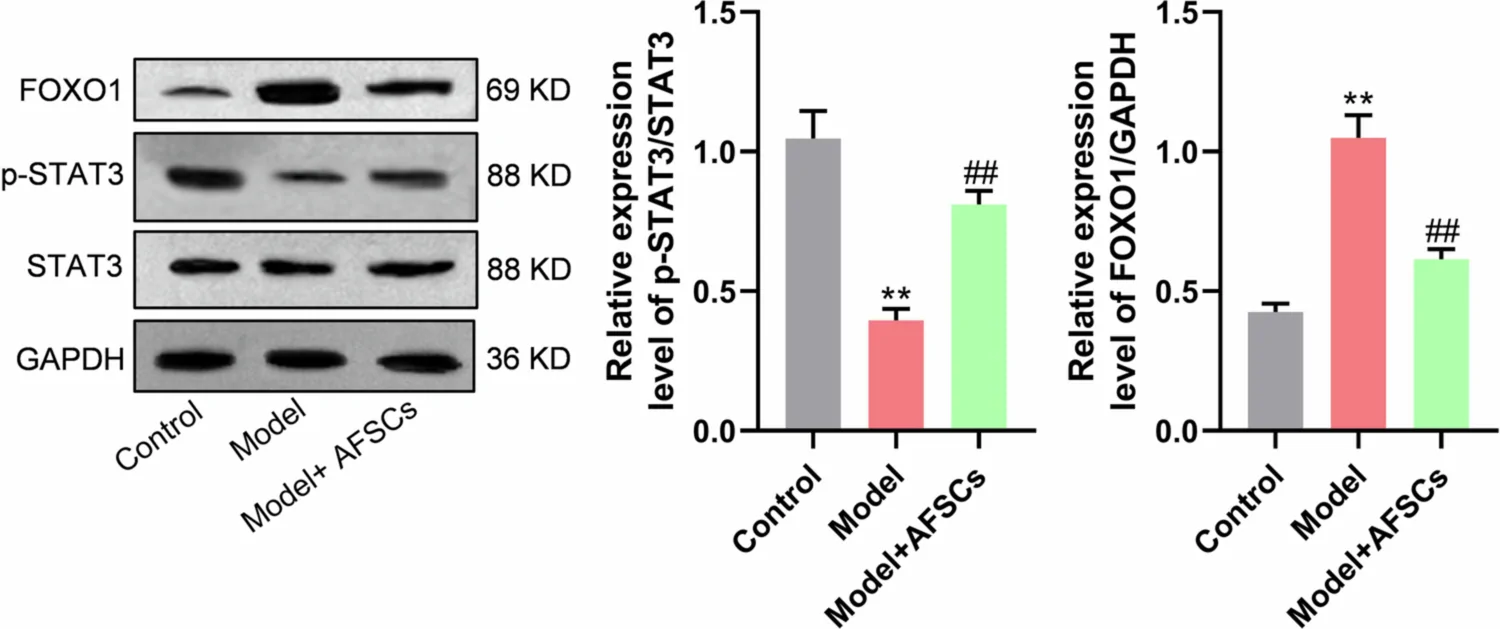
AFSCs regulated the FOXO1/STAT3 signaling in spinal cord tissues of SB rats. Animals were divided into three groups: Control, Model, and Model+AFSCs. Western blot analysis was utilized to determine levels of FOXO1 and p-STAT3/STAT3 in spinal cord tissues (**p < 0.01 vs. Control, ^##^ *p* < 0.01 vs. Model).

STAT3 is reported to positively regulate the progression of autophagy^23^ and FOXO1 reduces the STAT3 activation.^24^ Here, we found that FOXO1 levels were markedly elevated, while p-STAT3/STAT3 levels were notably repressed in spinal cord tissues of SB rats, which were strikingly rescued by the administration of AFSCs (Figure 3).

### AFSCs inhibited apoptosis and improved neuronal injury-related marker expression in M-NSCs isolated from SB rats

**Figure 4.**
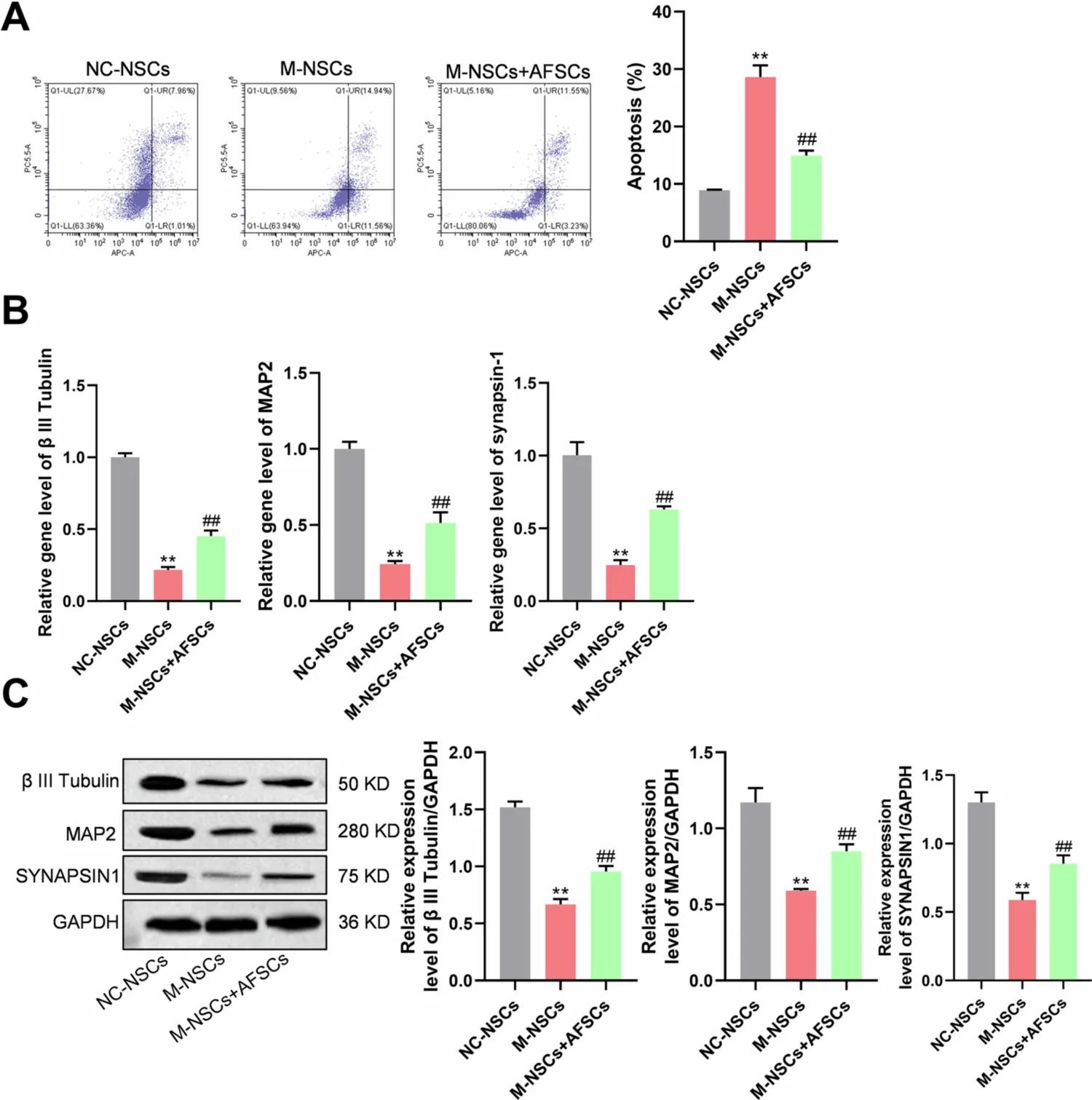
AFSCs inhibited apoptosis and improved neuronal injury-related marker expression in M-NSCs isolated from SB rats. M-NSCs were co-cultured with or without AFSCs, with NC-NSCs as the negative control. (A) Flow cytometry was used to determine the apoptotic rate of NSCs. (B) Gene levels of β III Tubulin, MAP2, and SYNAPSIN1 in NSCs were detected by RT-PCR. (C) Protein levels of β III Tubulin, MAP2, and SYNAPSIN1 in NSCs were determined by Western blot analysis (**p < 0.01 vs. NC-NSCs, ^##^ *p* < 0.01 vs. M-NSCs).

Firstly, NC-NSCs and M-NSCs were isolated and identified using the immunofluorescence detection of Nestin, with PC12 cells as the negative control (Figure S3). To evaluate the influences of AFSCs on NSCs, a co-culture system was established, with three groups divided: NC-NSCs, M-NSCs, and M-NSCs+AFSCs. The apoptotic rate of NC-NSCs was 4.79%, while the apoptotic rate of M-NSCs was 33.32%, which was notably reduced to 14.58% by AFSCs (Figure 4A). Furthermore, comparing to NC-NSCs, expressions of β III Tubulin, MAP2, and SYNAPSIN1 were strikingly repressed in M-NSCs, which were remarkably elevated by AFSCs (Figure 4B and C).

### AFSCs triggered autophagy in M-NSCs isolated from SB rats

**Figure 5.**
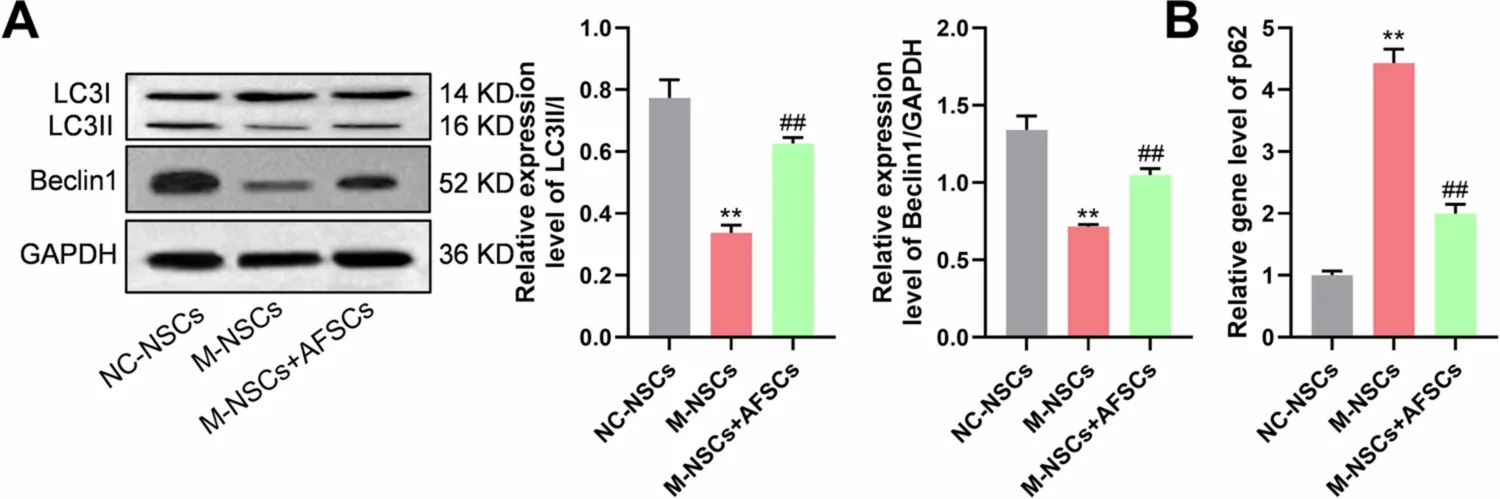
AFSCs triggered autophagy in M-NSCs isolated from SB rats. M-NSCs were co-cultured with or without AFSCs, with NC-NSCs as the negative control. (A) Western blot analysis was utilized to determine levels of LC3II/I and Beclin1 in NSCs. (B) Gene level of p62 in NSCs was detected by RT-PCR (**p < 0.01 vs. NC-NSCs, ^##^ *p* < 0.01 vs. M-NSCs).

Comparing to NC-NSCs, LC3II/I and Beclin1 expressions were sharply restrained in M-NSCs, which were strikingly enhanced by AFSCs (Figure 5A). Consistently, p62 expression was increased in M-NSCs and was reduced following co-culture with AFSCs (Figure 5B), providing additional evidence that AFSCs regulated autophagy-associated changes in M-NSCs.

### AFSCs regulated the FOXO1/STAT3 signaling in M-NSCs isolated from SB rats

**Figure 6.**
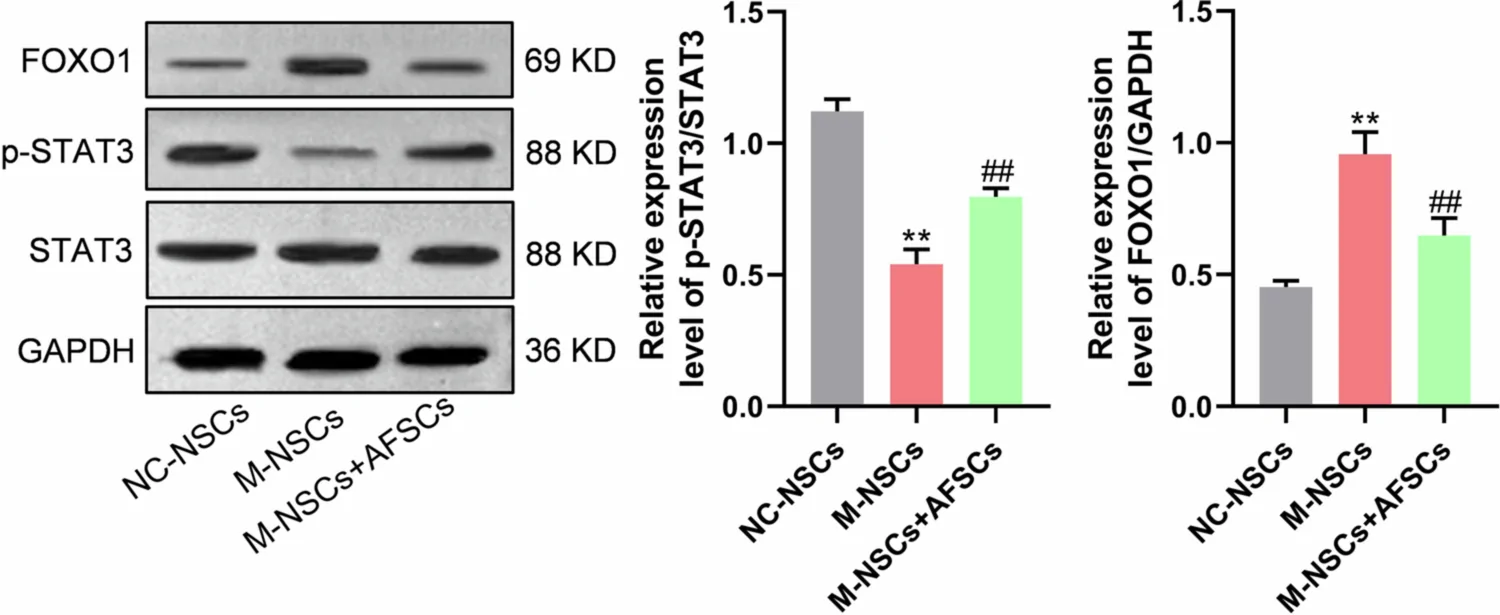
AFSCs regulated the FOXO1/STAT3 signaling in M-NSCs isolated from SB rats. M-NSCs were co-cultured with or without AFSCs, with NC-NSCs as the negative control. Western blot analysis was utilized to determine levels of FOXO1 and *p*-STAT3/STAT3 in NSCs (**p < 0.01 vs. NC-NSCs, ^##^ *p* < 0.01 vs. M-NSCs).

Comparing to NC-NSCs, FOXO1 was sharply upregulated, while p-STAT3/STAT3 were strikingly downregulated in M-NSCs, the levels of which were noticeably reversed by AFSCs (Figure 6).

### FOXO1 overexpression, STAT3 inhibition, and siRNA-mediated pathway modulation regulated influences of AFSCs on neuronal injury-related markers and autophagy in M-NSCs isolated from SB rats

**Figure 7.**
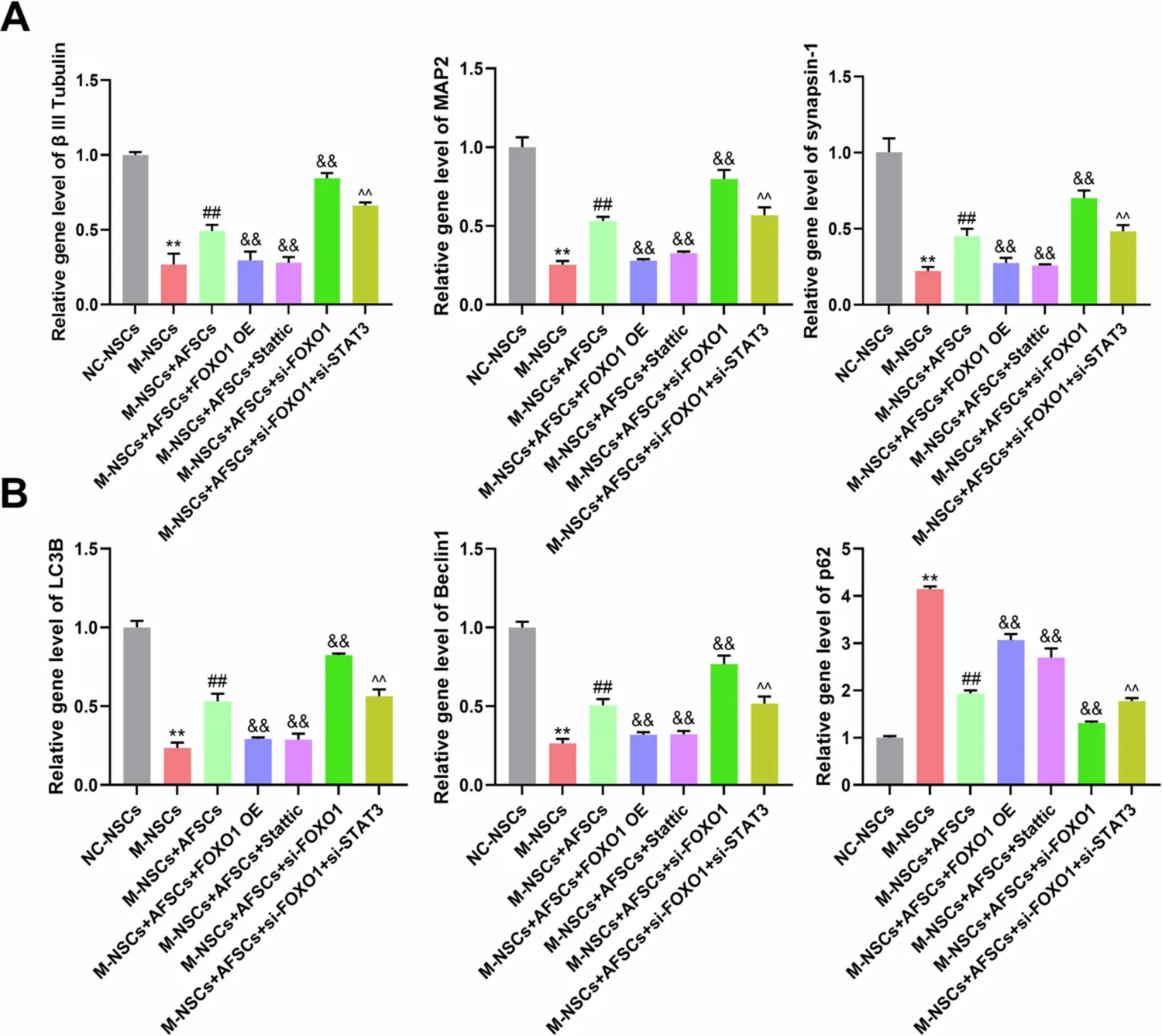
FOXO1 overexpression, STAT3 inhibition, and siRNA-mediated pathway modulation regulated influences of AFSCs on neuronal injury-related markers and autophagy in M-NSCs isolated from SB rats. FOXO1-overexpressed M-NSCs were co-cultured with AFSCs, M-NSCs were co-cultured with AFSCs in the presence of a STAT3 inhibitor (5 μM, Stattic), or M-NSCs were co-cultured with AFSCs after transfection with si-FOXO1 alone or combined si-FOXO1 and si-STAT3. (A) Gene levels of β III Tubulin, MAP2, and SYNAPSIN1 in NSCs were detected by RT-PCR. (B) Gene levels of LC3B, Beclin1, and p62 in NSCs were detected by RT-PCR (**p < 0.01 vs. NC-NSCs, ^##^ *p* < 0.01 vs. M-NSCs, ^&&^ *p* < 0.01 vs. M-NSCs+AFSCs, ^^*p* < 0.01 vs. M-NSCs+AFSCs+si-FOXO1).

M-NSCs were transfected with adenovirus containing FOXO1 OE for 48 h to establish FOXO1-overexpressed M-NSCs, and additional siRNA-mediated knockdown of FOXO1 or STAT3 was performed to further validate pathway involvement. The efficiency of FOXO1 overexpression, FOXO1 knockdown, and STAT3 knockdown was confirmed by RT-PCR (Figure S4). To confirm that FOXO1/STAT3 signaling is involved in the molecular effects of AFSCs, FOXO1-overexpressed M-NSCs were co-cultured with AFSCs, or M-NSCs were co-cultured with AFSCs in the presence of an STAT3 inhibitor (5 μM, Stattic). In parallel, M-NSCs were co-cultured with AFSCs after transfection with si-FOXO1 alone or combined si-FOXO1 and si-STAT3. Comparing to NC-NSCs, β III Tubulin, MAP2, and SYNAPSIN1 levels, as well as LC3II/I and Beclin1 expressions, were strikingly reduced in M-NSCs, which were notably enhanced by AFSCs. Following FOXO1 overexpression or co-culturing with Stattic, β III Tubulin, MAP2, and SYNAPSIN1 levels and LC3II/I and Beclin1 expressions were remarkably repressed, whereas p62 expression was increased. Conversely, FOXO1 knockdown further enhanced β III Tubulin, MAP2, SYNAPSIN1, LC3B, and Beclin1 expression and reduced p62 expression in AFSC-treated M-NSCs. Concurrent STAT3 knockdown partially attenuated the effects observed after FOXO1 silencing, including reduced neuronal marker and autophagy-related gene expression and increased p62 expression compared with si-FOXO1 alone (Figure 7A and B). These results provided additional genetic evidence supporting the involvement of the FOXO1/STAT3 axis in AFSC-mediated regulation of neuronal injury-related markers and autophagy-associated changes.

## Discussion

βIII Tubulin is specifically expressed in neurons as an isoform of tubulin, and it plays a key role in the organization, stability, and function of microtubules in neural cells.^25^ It is not only involved in the formation of microtubules but also serves as one of the neuronal markers, widely applied in neurobiological research.^26^ MAP2 is the main cytoskeleton regulatory protein in neuronal dendrites, affecting microtubule dynamics and microtubule/actin interactions, controlling the growth of neurites and synaptic function.^27^ The phosphorylation status of MAP2 can regulate its connection with the cytoskeleton and is regulated by development, potentially playing an important role in the growth of neuronal processes, synaptic plasticity, and neuronal cell death.^28^ SYNAPSIN1 is a synaptic protein whose expression and function are regulated by SIRT1, which can significantly inhibit the expression of SYNAPSIN1 and affect the growth of neuronal dendrites.^29^ NGFs are crucial in regulating spinal cord neural function, and they are known to stimulate the proliferation of NSCs, which is vital for the recovery of neural function following spinal cord injury.^30^ The addition of BBB locomotor scoring provided behavioral evidence that AFSC-treated SB rats displayed partial improvement in hindlimb locomotor performance during the observation period. This functional readout complemented the molecular findings; however, the improvement should be interpreted cautiously because BBB scoring mainly reflects open-field locomotor performance and does not fully capture sensory function, bladder function, electrophysiological conduction, or long-term neurological outcomes. Consistent with previous studies by Ma^31^ and Wei,^32^ in the spinal cord tissues of SB rats, apoptosis was noticeably induced, accompanied by downregulated β III Tubulin, MAP2, and SYNAPSIN1 levels, which were remarkably reversed by AFSCs, implying that AFSCs exerted protective effects on neuronal injury-related molecular changes in the spinal cord tissues of SB rats. Moreover, the reduced productions of NGFs in the spinal cord tissues of SB rats were remarkably enhanced by AFSCs, which further supported the potential neuroprotective effects of AFSCs in SB. Furthermore, apoptosis was activated in NSCs isolated from the spinal cord tissues of SB rats, along with downregulated β III Tubulin, MAP2, and SYNAPSIN1 levels, which were strikingly rescued by AFSCs, implying that NSCs might be one of the cellular targets through which AFSCs alleviate neuronal injury-related changes during SB.

Autophagy is a key mechanism for cells to maintain homeostasis and respond to stress, and it protects cells by clearing damaged organelles and protein aggregates.^33^ In the process of autophagy, LC3-II/I and Beclin1 play crucial roles. LC3-II is a key marker in the autophagy process, which is converted from LC3-I through a lipidation reaction during the formation of autophagosomes, a process that is essential for the maturation and degradation of autophagosomes.^34^ Beclin1 is another core protein in the autophagy process, which forms a complex with VPS34 and VPS15/p150, a complex that plays a vital role in the formation of autophagosomes.^35^ Aligned with the research claimed by Zhao,^36^ autophagy was noticeably inhibited in the spinal cords of SB rats, which was sharply activated by AFSCs, suggesting that AFSCs might alleviate neuronal injury-related alterations in the spinal cord tissues of SB rats via inducing autophagy. In addition, repressed autophagy in NSCs isolated from the spinal cord tissues of SB rats was improved by AFSCs, which further identified that AFSCs modulated neuronal injury-related molecular changes in SB rats via inducing the autophagy of NSCs.

STAT3 is a critical signaling molecule that plays a dual role in the regulation of autophagy, depending on its subcellular localization.^37^ Nuclear STAT3 can fine-tune autophagy by transcriptionally regulating autophagy-related genes such as BCL2 family members and BECN1. In contrast, cytoplasmic STAT3 inhibits autophagy by sequestering EIF2AK2 and interacting with other autophagy-related signaling molecules like FOXO1 and FOXO3.^38^ FOXO1 is a transcription factor that plays a crucial role in cellular processes, including apoptosis and autophagy.^39^ It has been shown to negatively regulate STAT3 activation by directly interacting with it, thereby preventing STAT3 from binding to its target genes.^40^ Herein, both in spinal cord tissues and NSCs isolated from the spinal cord tissues of SB rats, STAT3 signaling was repressed, while FOXO1 was upregulated, implying that neuronal damage in the spinal cords of SB rats might be mediated by FOXO1/STAT3 axis mediated autophagy inhibition. The administration of AFSCs dramatically reversed the inactivated STAT3 signaling and the upregulated FOXO1 in both in spinal cord tissues and NSCs isolated from the spinal cord tissues of SB rats, indicating that AFSCs might trigger autophagy in NSCs via regulating the FOXO1/STAT3 axis. Furthermore, the influences of AFSCs on neuronal injury-related markers and autophagy in NSCs isolated from SB rats were significantly abolished by FOXO1 overexpression or STAT3 inhibition, which further identified that AFSCs alleviated neuronal injury-related molecular changes in SB rats by inducing autophagy in NSCs via regulating the FOXO1/STAT3 axis. The additional gene-silencing experiments further strengthened this mechanistic interpretation. FOXO1 knockdown enhanced the AFSC-associated upregulation of neuronal markers and autophagy-related genes, whereas simultaneous STAT3 knockdown partially reversed the effects associated with FOXO1 silencing. These findings are consistent with a model in which FOXO1 acts upstream of STAT3 in this context, and AFSCs may promote neuroprotective molecular responses at least partly by suppressing FOXO1 and restoring STAT3-associated autophagy regulation. In addition, p62/SQSTM1 was assessed as an additional autophagy-associated marker. Increased p62 expression in SB spinal cord tissues and M-NSCs, together with reduced LC3II/I and Beclin1, suggested impaired autophagy-associated activity, whereas AFSC treatment reduced p62 and increased LC3II/I and Beclin1. Although these data provide broader support for AFSC-mediated autophagy modulation, p62 expression alone does not fully define autophagic flux; therefore, future studies using lysosomal inhibitors, tandem fluorescent LC3 reporters, and direct autophagic flux assays will be required to more precisely characterize dynamic autophagy activity. In future studies, the specific factors carried by AFSCs that contribute to their neuroprotective molecular effects in SB will be further explored. Although this study identified the FOXO1/STAT3-autophagy axis as an important mechanism by which AFSCs alleviated SB-related neuronal injury, other molecular pathways or proteins that were not examined here may also participate in this process. For example, glial cell line-derived neurotrophic factor (GDNF) has been implicated in neuroprotection, synaptic remodeling, and neural regeneration after spinal cord injury, and altered plasma GDNF levels have also been reported in patients with SB, possibly reflecting the biological consequences of intrauterine spinal cord exposure.^41^ In addition, oxidative stress may contribute to fetal tissue injury in SB. Because the fetal antioxidant defense system is relatively immature, excessive reactive oxygen species (ROS) can induce protein, lipid, and DNA damage, thereby promoting apoptosis or necrosis in vulnerable neural tissues.^42^ Autophagy is also closely linked to mitochondrial quality control. The WIPI protein family and PINK1/Parkin-mediated mitophagy pathway may be relevant to neuronal homeostasis during neural tube closure and neural development, suggesting that mitochondrial autophagy may represent another mechanism involved in SB-related neural injury and repair.^43^ ^,^ ^44^ Moreover, epigenetic regulation, including microRNAs and DNA methylation, has been associated with altered expression of SB risk-related genes and disruption of normal neural tube development.^45^ ^,^ ^46^ Therefore, future studies should investigate whether GDNF signaling, oxidative stress-responsive factors such as Nrf2, mitophagy-related proteins, and epigenetic regulators interact with the FOXO1/STAT3 axis and jointly regulate the neuroprotective effects of AFSCs in SB.

Several limitations should be acknowledged. First, this study was performed in a preclinical neonatal rat model of SB, which cannot fully reproduce the anatomical complexity, fetal surgical timing, maternal–fetal environment, and clinical heterogeneity of human SB/MMC. Therefore, the translational relevance of direct intraspinal AFSC administration requires further validation in larger animal models that more closely resemble the human spinal defect and the current prenatal repair setting. Second, although postnatal intraspinal injection allowed precise delivery of AFSCs to the lesion-centered spinal cord region in this experimental model, this strategy should be interpreted as an exploratory local cellular approach and a potential adjunct to anatomical defect closure rather than a replacement for established prenatal surgical repair. At present, prenatal repair remains the clinically established intervention known to improve selected outcomes in appropriately selected SB/MMC cases, whereas direct spinal cord injection of AFSCs has not been validated in a fetal surgical setting. Therefore, the current data should not be interpreted as supporting the immediate clinical application of AFSC injection. Instead, they provide mechanistic evidence that may inform future studies evaluating whether local AFSC delivery can enhance neuroprotective molecular responses when combined with anatomical closure. Third, the present study focused mainly on molecular and cellular endpoints, including apoptosis-related markers, neuronal markers, nerve growth factor expression, autophagy-related proteins, and FOXO1/STAT3 signaling. BBB locomotor scoring provided an initial behavioral assessment and showed partial improvement in hindlimb locomotor performance in AFSC-treated SB rats. However, BBB scoring alone does not establish comprehensive neurological recovery because sensory responses, bladder function, electrophysiological conduction, gait parameters, and long-term neurological outcomes were not evaluated. Therefore, the current findings should be interpreted as evidence of neuroprotective molecular effects accompanied by partial locomotor improvement rather than definitive functional neurological repair. The use of random assignment and blinded behavioral/quantitative assessment strengthened the experimental control; nevertheless, the relatively small number of animals in each group may limit the statistical power and generalizability. Larger independent cohorts will be needed to confirm the robustness of the behavioral and molecular findings. Fourth, spinal cord tissues were collected 14 d after treatment, and longer follow-up is required to determine the durability of AFSC effects, cell survival, biodistribution, possible ectopic differentiation, immune responses, and delayed safety events. Finally, autophagy-associated changes were evaluated using LC3II/I and Beclin1 protein levels together with p62 mRNA expression; although this expanded marker panel improved the assessment of autophagy-related molecular alterations, it still did not directly measure p62 protein turnover or dynamic autophagic flux. Future studies using lysosomal inhibition, tandem fluorescent LC3 reporters, in vivo pathway-specific modulation, and broader screening of neurotrophic, oxidative stress-related, mitophagy-related, and epigenetic regulators will be needed to further define the causal mechanisms underlying AFSC-mediated neural repair. Accordingly, the terms “repair of neurological function” and “functional recovery” should be interpreted cautiously in the context of the present study, as the data mainly reflect changes in molecular and cellular markers associated with neuronal injury, survival, autophagy, and neuroprotection. Substantial translational barriers remain before any human application can be considered. Direct spinal cord delivery of AFSCs would need to be tested in larger fetal models that more closely approximate human SB/MMC anatomy, lesion size, tissue fragility, and the operative environment of prenatal repair. The delivery route, injection depth, cell dose, timing relative to anatomical closure, and feasibility of combining local cell delivery with established fetal surgical repair would require careful optimization. Long-term studies are also needed to evaluate cell survival, biodistribution, persistence within the lesion, possible migration outside the target region, ectopic differentiation, immune responses, tumorigenicity, and delayed adverse events. In addition, standardized cell isolation, expansion, quality control, potency testing, and good manufacturing practice-compatible production would be necessary before considering any clinical protocol. Therefore, the present findings should be regarded as preclinical mechanistic evidence that informs future experimental work rather than evidence of readiness for human application.

Taken together, AFSCs alleviated neuronal injury-related changes in SB rats by inducing autophagy via regulating the FOXO1/STAT3 axis, accompanied by partial improvement in BBB locomotor scores, which provides preclinical evidence for an experimental neuroprotective mechanism in SB. Further studies in clinically relevant prenatal repair models with long-term safety and comprehensive functional outcome assessments are required before translational applicability can be considered.

## Supplementary Material

Supplementary material Fig S1.jpg

Supplementary material Fig S2.jpg

Supplementary material Fig S3.jpg

Supplementary material Fig S4.jpg

Supplementary material Supplementary figure caption

## Data Availability

Data will be available by request to the corresponding author.
