# Supplementary material for: Amniotic fluid stem cells alleviate neuronal injury-related changes in spina bifida by inducing autophagy via regulating the FOXO1/STAT3 axis
Source: Organogenesis. 2026 Sep 11;22(1):2728258. doi: 10.1080/15476278.2026.2728258 (PMC13577246; doi:10.1080/15476278.2026.2728258)
Supplement: Supplementary file 1 [file KOGG_A_2728258_SM3531.docx]

Fig S1. Identification of isolated AFSCs. The isolated AFSCs were identified by determining expressions of CD45, CD90, and OCT4 using the flow cytometry.

Fig. S2. AFSCs promoted nerve growth factor production in spinal cord tissues of SB rats. Animals were divided into 3 groups: Control, Model, and Model+AFSCs. Gene levels of Ngf, Egf, Igf, Fgf2, Fgf10, Bdnf, and Tgf-β in spinal cord tissues were detected by RT-PCR (**p<0.01 vs. Control, ##p<0.01 vs. Model).

Fig. S3. Identification of isolated NSCs. NC-NSCs and M-NSCs were isolated and identified using immunofluorescence detection of Nestin, with PC12 cells serving as the negative control.

Fig S4. RT-PCR validation of FOXO1 overexpression and siRNA-mediated knockdown efficiency. A. Gene level of FOXO1 in M-NSCs transfected with FOXO1 OE or OE-NC was detected by RT-PCR. B. Gene level of FOXO1 in M-NSCs transfected with si-FOXO1 or si-NC was detected by RT-PCR. C. Gene level of STAT3 in M-NSCs transfected with si-STAT3 or si-NC was detected by RT-PCR (**p<0.01 vs. OE-NC or si-NC).
